# Access to public health care and associated factors in rural areas: a cross-sectional community-based study in Bopa district, Southern Benin

**DOI:** 10.11604/pamj.2024.49.46.42825

**Published:** 2024-10-18

**Authors:** Landry Assongb, Marius Ouendo, Martin Akogbeto, Edouard Dangbenon, Achille Massougbodji, Jackie Cook, Manfred Accrombessi

**Affiliations:** 1 Institut de Recherche Clinique du Bénin (IRCB), Abomey-Calavi, Benin,; 2 Centre de Recherche Entomologique de Cotonou (CREC), Ministère de la Santé, Cotonou, Benin,; 3 Institut Régional de Santé Publique, Université d´Abomey-Calavi, Ouidah, Benin,; 4 Medical Research Council (MRC) International Statistics and Epidemiology Group, London School of Hygiene and Tropical Medicine, London, United Kingdom,; 5 Faculty of Infectious and Tropical Diseases, Disease Control Department, London School of Hygiene and Tropical Medicine, London, United Kingdom

**Keywords:** Health care access, health services, treatments access, health insurance, health facility

## Abstract

**Introduction:**

despite the considerable progress made to date, access to health care in public health facilities remains a challenging public health problem in Benin. This study aimed to assess trends in access to care over five years and to identify factors associated with low access to care.

**Methods:**

a cross-sectional community-based study was conducted in the Bopa district, a rural area of southern Benin between January and February 2020. Twenty (20) villages were randomly selected using the two-stage probabilistic clustering method. The sample consisted of 620 participants (31 per village) distributed across the seven sub-districts of the Bopa district. Mixed-effect logistic regression models, using a clustered sampling design, were used to identify the factors associated with low access to care at public health centers.

**Results:**

less than half of the recruited sick participants (38.9%) reported having had access to care at public health facilities in the month before the visit. Using public health services proportion in the population progressively increased from 2014 (29.7%) to 2019 (47.1%). Factors associated with no access to care were lack of mutual health insurance (adjusted odds ratio (aOR: 5.3, 95% CI: 2.1-13.5, p=0.001); low household income (aOR: 3.7, 95% CI: 1.7-8.1, p=0.001); and lack of transport (aOR: 3.4, 95% CI:1.8-6.2, p<0.001).

**Conclusion:**

this study highlights the importance of a well-implemented and sustained community-based mutual health insurance system, particularly in rural areas. In addition, improving the living standards of the population would likely increase access to care. Policy makers should take these factors into account.

## Introduction

Worldwide, more than 400 million people do not have access to basic health services and 40% live without any social health protection, according to reports by the International Labour Organization (ILO) and the World Health Organization (WHO) in 2014 and 2015 respectively [1]. Access to health care can be defined as the ease with which a population can obtain the health care services it needs. It is related to the potential presence (or absence) of economic, physical, cultural, geographical, or other barriers to the use of these services [2]. A study carried out in 2010 by the National Institute of Statistics (INS) of Cameroon, after analyzing the results of various data collected in more than 79 African countries around the world, formally shows that the health coverage rate in sub-Saharan Africa had never exceeded 50% [3]. In its 2018 report, the WHO confirmed that access to essential health care and services is very low in all countries in the region, at 32% against a demand of 67% [4]. In Benin, despite the progress made so far in terms of health coverage and management of health facilities, the health coverage rate remained similar between 2010 and 2017 (46.8% and 47.6% respectively) [5,6]. By 2019, access to health services had increased to 59.1% in 2019 [7]. In some regions of the country, such as the Comé-Grand Popo-Houeyogbé-Bopa health zone, the rate of use of health services remains low. Between 2015 and 2017, the rate for this zone varied between 43.2% and 55.5%, with lower proportions inside the region [6,8].

Measures taken to improve this situation include the free treatment of certain diseases (malaria and malnutrition in children under five), treatment of the indigent with low incomes, strategy for the development of mutual health insurance, introduction of performance-based financing, suppression of the informal circulation of medicines and measures to regulate the practice of health professionals in private or public clients. Despite these measures, the utilization rate of health services is still low and the health insurance system has not been able to meet the needs of its beneficiaries. In the Comé-Grand Popo-Houéyogbé-Bopa health zone, the average healthcare attendance rate was 63.8% in 2019 [7]. Access to health care is one of the cornerstones of improving population health in the context of sustainable development goals. Health policies should therefore promote access to health services for all, regardless of socioeconomic status and geographical location. Unfortunately, a large proportion of the population, particularly in rural areas, remains without access to care. This study aims to assess trends in access to care over five years and identify factors associated with low access to care in rural areas of Benin. It was specifically to determine the utilization rate of health services in the commune of Bopa from 2015 to 2019 and identify factors associated with non-access to public health services in the event of illness. As a starting hypothesis, we estimated that the lack of financial means to cover healthcare needs is associated with non-access to public health services in the event of illness. Community data collection took place from January to February 2020.

## Methods

**Study design and setting:** a cross-sectional community survey was conducted between January and February 2020 to understand the use of public health centers for illness and the factors associated with access to care at public health facilities. Bopa is a commune in the south of Benin. It is made up of 60 villages spread over the 07 arrondissements that make up the commune. Each arrondissement has a public health centre and a population census (updated every year). The total number of inhabitants is 111150. The commune is crossed to the east by the Couffo River and Lake Ahémé, and the main activities carried out there are agriculture and fishing.

### Procedures

**Study population:** data from public health facilities were collected from annual 2014-2019 reports in the health district. Villages and households were randomly selected using the two-stage probabilistic clustering method. First, 20 clusters (villages) out of 60 were selected using a probability proportional to size sampling; and then 31 households were randomly selected from each cluster. The following criteria enabled us to include the patients in the study: be the household head or his/her representative, be at least 18 years old and reside permanently in the household in order to qualify as the head of household's representative. Have lived in the area for more than a year, and have given their free and informed consent to participate.

**Variable and data sources:** face-to-face interviews were conducted in community with the household head using a standardized questionnaire. The main outcome is the use or non-use of a healthcare facility during the last illness of a household member. The period considered is that of the last four weeks prior to the survey. Other data collected included household socio-demographic data (age of household head, gender, ethnicity, education, marital status, household size), socio-cultural data (perception of illness, religion, membership of a health mutual, social pressures), household socioeconomic data (household income, household head activity, mean´s transport possession), geographical access to health centres (duration, average distance, obstacles on the way), and data related to health centers (waiting time at the health center, quality of reception, satisfaction aftercare, perceptions of the cost of services and medication). Public health services use over the last five years was obtained from the 07 health facilities in the district. Access to health services was available in the annual statistical reports of each health center. The random sample used made it possible to limit potential selection bias. In order to limit memory bias, we limited the period to the last four weeks prior to the survey.

**Definitions:** access to health services was calculated for each center by dividing the total number of outpatients and inpatient visits received by the population covered by the centre for the year. Illness was defined as an alteration in the state of health requiring care (fever, road accident, vomiting, heat). For households with mutual health insurance, the reimbursement rate is the proportion of the total cost of care covered by the insurance in the event of illness.

**Size sample:** we collected data on the use of public health services from 2014-2019 in all 07 health centers in the municipality. Thus, 05 annual summaries were reviewed per health center. For the community data collection, data were collected from 620 people using the Schwartz formula with a risk of error of 5% and a correction factor of 1.5.

**Data management and statistical analysis:** poor access to public health facilities services was defined as all those who did not use the health center during their last illness (in the 4 weeks before the survey). Data collected were double-entered using epi-info 7.1.1.14 software and then analyzed using SPSS version 18.0. We used the average (SD) to present the quantitative variables and the proportion to present the qualitative variables. Pearson's chi-squared test was used to compare proportions. Mixed-effect logistic regression models were used to identify the factors associated with access to public health care centers, with clusters included as a random effect. Variables with P values below 0.2 were included in multivariate analyses and were eliminated step by step using the backward selection procedure. Only variables whose P < 0.05 were retained in the final model. For variables with more than two categories, a P value of the global test is given.

**Ethical considerations:** the study obtained approval from the Coordinating Committee of the Public Health Teaching and Research Unit of the University of Abomey-Calavi. It was also submitted for approval to the health director of the Mono-Couffo department. The study was conducted in accordance with the declaration of Helsinki. Informed consent was obtained from the head of the household or his/her representative.

## Results

**General characteristics of the study population:** a total of 620 households were surveyed. All the households selected were eligible and none refused to take part in the study. The majority of household heads were male (90%) and the average age of household heads was 43 years (±14). Most household heads were monogamous in marital status (60.8%) and the average household size was 6 (± 2.3) resident inhabitants. The predominant ethnic group was the Sahouè (89.4%). More than 70% of household heads were without education (77.4% of respondents). Only 10% of households were covered by a mutual health insurance scheme. For households that were not covered by mutual health insurance, the main constraint was a lack of financial income, reported by 70.1% of participants. For others (11.5%), this was considered as waste of money as they did not often use the health centres. Few households had transport means (30.8%). Regarding household income, 81.3% had a daily income of less than US$ 3.31 (Table 1). Almost all households surveyed (96.1 %) had at least one household member who had been ill in the four weeks prior to the survey. However, only 38.9% of them had visited a health facility. Malaria was the most commonly reported reason for feeling unwell (49.7%).

**Table 1:** general characteristics of the study population

General characteristics	N	%
Sex of head of household (N = 620)		
Male	558	90
Household head means age (N = 620) ; Means = 42.5 ≈43 +/-14		
Marital statut (N = 620)		
Monogamous	377	60.8
Polygamous	168	27.1
Other	75	12.1
Household size (N = 620) ; Means = 5.53 ≈6 +/-2.3		
Ethnic (n = 620)		
Sahouè	554	89.4
Other	66	10.6
Education level (N = 620)		
No educate	480	77.4
Current membership of a mutual health insurance company (N = 620)		
Yes	62	10
Reason for non-membership of mutuals (N = 558)		
Financial difficulties	391	70
Waste/non-use of health centre	64	11.5
Other	103	18.5
Owning means of transport (N = 620)		
Yes	191	30.8
Daily income (N = 620)		
< US$3.31	504	81.3
In the last 4 weeks, at least one member has been ill (N= 620)		
Yes	596	96.1
Use of public health services for this disease (N= 596)		
Yes	232	39
Reason for consultation/use of public health service (N= 596)		
Malaria	296	49.7
Respiratory disease	88	14.8
Diarrhoeal disease	78	13.1
Other	159	27
First-line treatment for this disease (N= 596)		
Health centre	88	14.8
Self-medication (modern and traditional)	500	83.9
Autres	8	2
Second-line treatment if not satisfied in first-line treatment (N= 249)		
Health centre	132	53
Other	117	47
Lack of money to go to the health centre when sick (N= 620)		
Yes	616	99
Median time walking from homes ; Median= 68 minutes (IQR=70)		
Median health centre waiting time; Median= 17 minutes (IQR=20)		
Natural obstacles on the road (N= 620)		
Yes	275	44
Practicability of routes (N= 620)		
No	379	61

**Figure 1:**
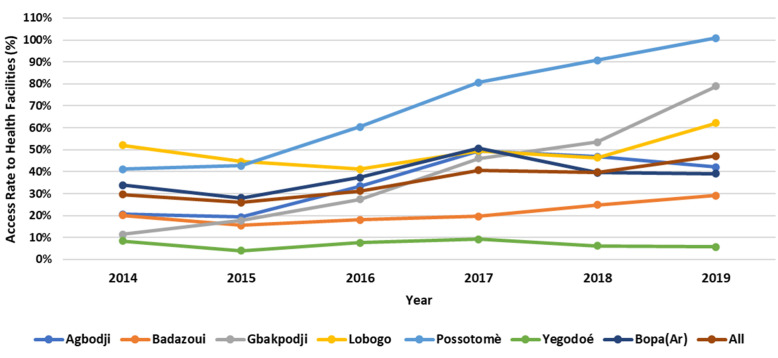
trends in attendance rates at district health centres from 2014 to 2019, based on monthly data from health centres in Bopa’s district

In the case of illness, self-medication (modern or traditional) was the first line of treatment for the majority (89.8%). Attending a health facility was considered in the second option (53.0%) when there was no recovery with the self-medication. Financial means were the main challenge reported for attending a health facility (99.4% of respondents) (Table 1). The median time spent walking from participants´ homes to the health facilities was 68 minutes (IQR= 70) with a median waiting time of 17 minutes (IQR= 20) once at the health facility. Regarding geographical accessibility, 61.1% lived in areas with poor road access, especially during rainy seasons, and 44.4% were in areas with natural obstacles in the way (river, forest, etc.) (Table 1). Overall, the healthcare access rate has increased from 29.7% in 2014 to 47.1% in 2019. In most health facilities, access to care remained low and constant between 2014 and 2019. There was, however, a substantial increase in the Gbakpodji and Possotomè health sub-district facilities (Figure 1).

**Factors associated with access to public health care:** in Table 2 presents factors associated with access to public health care. Household status, household membership of a mutual health insurance scheme, household size, ownership of a means of transport, average daily household income, decision-maker regarding the choice of treatment, availability of medicines at the health centre, spiritual perception of illness and satisfaction with the care received at the health centre in previous visits were factors significantly associated with access to health care after univariate analysis (Table 2). In multivariate analysis, households without mutual health insurance were five times more likely not to seek health care when ill (adjusted odds ratio aOR 5.3, 95% CI: 2.1-13.5, p=0.001). Households with a daily income of less than US$3.31 were also three times more likely not to attend public health services (aOR: 3.7, 95% CI: 1.7-8.1, p=0.001). Households with no means of transport were three times more likely not to access public health services (aOR: 3.4, 95% CI: 1.8-6.2, p<0.001) (Table 3).

**Table 2:** relationship between the independent variables and access to the health centre despite being ill in the month prior to the survey: Bopa district in 2020: univariate analysis

	Access to health centres when sick				
Characteristics	n/N	(%)	OR	95% CI	P-Value
Marital Status of household head					
Single/ Divorced/ Widowed	19/596	27,9%	1		
Married/In couple	213/596	40,3%	1,74	0,99 – 3,04	0,048
**Households with health insurance**					
Yes	52/596	83,9%	1		
No	180/596	33,7%	10.23	5,07– 20,6	<0,001
**Possession of transportation means in household**					
Yes	152/596	71,4%	1		
No	100/596	24,3%	7.74	5,24 – 11,44	<0,001
**Average daily income in household**					
≥ US$3.31	90/596	81,1%	1		
< US$3.31	142/596	29,3%	0.09	0,05 – 0,16	<0,001
**Unavailability of services and medicines in health centre**					
No	72/596	30,1%	1		
Yes	160/596	44,8%	1.88	1,33 – 2,66	<0,001
**Disease deemed spiritual by household head**					
Yes	140/596	44,6%	1		
No	92/596	32,6%	0.60	0,43 – 0,84	0,003
**Post-care satisfaction in health centre**					
Yes	162/596	41,9%	1		
No	70/596	33,5%	1.43	1,00 – 2,03	0,046
**Household size**					
< 6	141/596	42,5%	1		
≥ 6	91/596	34.5%	1.40	1,00 – 1,96	0,047

**Table 3:** variables associated with not access to health centre despite being ill in the month prior to the survey, Bopa district in 2020: multivariate analysis

Characteristics	aOR	95% CI	P-value
Current membership in a mutual health insurance company			
Yes	1	2.05 – 13.47	0.001
No	5.26		
**Average daily household income**			
< US$3.31	1	1.68 – 8.12	0.001
≥ US$3.31	3.69		
**Means of travel**			
Yes	1		
No	3.38	1.83 – 6.24	<0.001

## Discussion

This study aimed to investigate the factors associated with low access to health care in the district of Bopa, a rural area in Benin. In this study, approximately 96% of respondents reported that at least one household member had been sick in the 4 weeks before the survey. However, only 38.9% of them had used public health services for care management. These results confirmed the low access to public health services in this community. These results are similar to those of Simaga K in Bamako in 2017 among heads of households, where 36.9% had used curative care [9]. The definition of access to health services, according to other authors, is sometimes defined as having used public health care at least once in the past. This definition does not necessarily make it possible to identify access to public health care over a given period and, in particular, to assess current access to public health care. This study found that households without health insurance at the time of the survey were less likely to use public health care services than households with mutual health insurance (33.7% vs. 83.9%). This finding was also reported in 2011 in the Karisimbi region of Congo (86.1% of mutual members used health care services compared to 42.1% of non-mutual members) [10]. In an unfavourable economic environment, it is difficult for the population to cope with the cost recovery mechanism implemented, which is mainly based on direct payments for care received [11-13].

According to a similar study conducted in Mali in 2008, members of mutual insurance companies who were up to date with their contributions, were two times more likely to be treated for fever at a health facility, and three times more likely to take their child suffering from diarr hoea to a health facility [14]. Socioeconomic status plays an important role in access to health care [15-18]. In our study, we found that households with a daily income of less than US$ 3.31 were three times more likely to avoid going to a health centre when feeling ill. Ouendo *et al*. have also reported a strong association between socioeconomic level and the use of public health services in a study conducted in Benin on the therapeutic itinerary of indigent patients [19]. This observation is supported by the 2008 US study on poverty and access to health care in developing countries. They concluded that the poor suffer a disproportionate burden of disease and generally have less access to health care, whether in terms of geographical accessibility, availability or affordability [20]. Indigent patients are less likely than non-indigent patients to use public health services when seeking care for the first time. Suzuki *et al*. have also observed the same result in their study on the characteristics of sudden and unexpected cancer deaths investigated by medical examiners in Tokyo. They showed that among the recorded deaths, indigent people were the largest subgroup (43%) among those who had never received medical advice [21].

This situation reflects the poor access of the indigent to health care, resulting in late diagnosis of the disease [21,22]. However, modern and traditional self-medication were the most commonly used first-line methods by the population [15]. These results are similar to ours, with only 14.8% of the population using public health services as their first source of care, compared to 83.9% who preferred self-medication (modern or traditional). The 2018´s WHO report on the state of health in the African Region, has supported the link between income and the use of public health services [4]. In a context where reimbursement is based on direct payments, people's purchasing power is important to ensure they have the resources to meet their health needs. Access to healthcare might also require significant investment, not only in terms of the availability of services and staff, which requires significant investment but also in the development and effective monitoring of operating models to optimise outcomes [4]. Households without means of transport were less likely to access health facilities than those with means of transport and were three times more likely to be unable to access public health services in the event of illness. These findings are similar to those of Olvera *et al*. on transport to health centres as a major barrier to health services [23]. Households close to health centres walk to them (38% in Conakry and 35% in Douala), while those further away who have no means of transport have to wait for public transport, either by tricycle or bush taxi. The studies conducted by Syed *et al*. and Marrocco *et al*. confirms that transport barriers are a major obstacle to accessing healthcare, particularly for people with low incomes or who are uninsured or underinsured [24,25].

In our study, we found that self-medication was the first choice of 83.9% of households in the event of illness. This was also observed by a study conducted in southern Benin with 88% of self-medication (either infusions (herbal teas) or modern medicines), confirming our findings [26]. The study on the Factors determining the low use of curative care services in the Karisimbi health zone, found that none of those who used natural medicines when they were ill-attended a health centre [10]. It should also be noted that in our study, 99.7% of those questioned agreed with the effectiveness and availability of plants for medical treatments. Other factors are also known to influence access to health care. Having friendly and welcoming health workers is often associated with a high use of health services [9,27,28]. In our study, there was no statistical difference between a good welcome and access to public health services. However, more than half of the respondents reported poor reception as the main reason for not using public health services when they were ill, and the health facilities with poor reception tended to also have low attendance rates. Most studies have shown that satisfaction with health care is mainly a function of the quality of reception and interpersonal relationships [29,30].

Our study has some limitations that should be considered. First, the study was cross-sectional and there is less evidence to establish causality relationships. The COVID-19 pandemic has certainly affected access to health services for the African population [31]. However, this study was conducted between January and February 2020, a few weeks before the report of the first confirmed case in Benin (March 2020). The definition of illness is subjective and could be subject to bias. For this reason, we ensured that respondents understood the concept of illness as a change in their state of health requiring care. Another limitation of this study is that we were not able to collect certain information about the ill person. It may be that the decision to use the health centre in case of illness depends on the sex of the sick person (male or female) or the age of the person (child or adult). Mixed research including quantitative and qualitative approaches, would be a better design to understand the low access to health services.

## Conclusion

Public health services in Benin are poorly used by the population. Low average household income, lack of a mutual health insurance scheme and lack of transport were the main factors significantly associated with poor access to public health services. Distance from health centres and poor reception at health facilities were also contributing factors. This study highlights the importance of a well-structured and sustainable community-based mutual system in improving the standard of living of the population.

### What is known about this topic

- The population’s poor access to public health care is well known, but less documented in Benin;
- Several studies have examined the factors that hinder the specific use of certain health services, but few studies have assessed the factors associated with access to health services in general.

### What this study adds

- Emphasise the importance of a well-structured and sustainable community health insurance system in improving access to public health care services;
- Identify factors contributing to poor access to health care services and the burden of self-medication. It would therefore be important to address the issue by seeking to strengthen and channel traditional medicine where necessary;
- Improve the living conditions of the population to enable them to have a minimum income to meet their basic needs.
